# Mitro-aortic pathology: a point of view for a fully transcatheter staged approach

**DOI:** 10.1007/s12471-017-1028-6

**Published:** 2017-08-02

**Authors:** G. D’Ancona, L. Paranskaya, A. Öner, S. Kische, H. Ince

**Affiliations:** 1 Heart Center, Rostock University Hospital, Rostock, Germany; 2 Department of Cardiology, Vivantes Klinikum im Friedrichshain und Am Urban, Berlin, Germany

**Keywords:** Aortic stenosis, TAVI, Mitral insufficiency, MitraClip double valve

## Abstract

Severe aortic valve stenosis (AVS) and mitral valve regurgitation (MVR) often coexist. Although a fully percutaneous treatment for the two conditions, by means of transcatheter aortic valve implantation (TAVI) followed by MitraClip, can be appealing in selected high-risk candidates, critical and strategical reasoning should be applied. In a 3-year period we have developed a single-centre experience of 14 patients who were managed with a staged percutaneous approach to treat severe AVS and MVR. The average interval from TAVI to MitraClip repair was 101 ± 12 days. Success for TAVI was 100% and 92.9% (13/14) for MitraClip. At late follow-up, 3 patients developed MVR 3+. Estimated 1‑year survival was 66.5%. Freedom from 1‑year endpoint (death, stroke, major bleeding, myocardial infarction, and cardiac re-hospitalisation) was 57.9%.

In our view, a fully transcatheter approach for mitro-aortic pathology is feasible and should be performed only as a staged procedure in those patients that remain symptomatic, in spite of successful TAVI. It should be emphasised that although the periprocedural success rate is satisfactory, follow-up mortality and re-hospitalisation rates remain high, even at mid-term follow-up. This most probably results from the advanced clinical picture at time of referral for treatment.

## Introduction

Severe aortic valve stenosis (AVS) and mitral valve regurgitation (MVR) are coexisting in 48% to 90% of candidates for conventional aortic valve replacement and transcatheter aortic valve implantation (TAVI) [1, 2]. A staged transcatheter approach to address first the aortic and then the mitral pathology may seem logical in patients with a complex comorbid profile. We present our single-centre experience with staged treatment of severe AVS and MVR by means of TAVI followed by MitraClip.

## Materials and methods

All data concerning patients treated at the Rostock University Hospital with TAVI and MitraClip were prospectively collected in a computerised database and Institutional Review Board approval for the study was obtained. The indication for treatment of mitral regurgitation and aortic stenosis was according to current guidelines and was discussed in an interdisciplinary heart team [3]. Surgical risk was assessed with the EuroSCORE and the Society of Thoracic Surgeons (STS) mortality risk calculation. Exclusion criteria were significant mitral stenosis and acute endocarditis. Only patients with severe aortic stenosis and mitral regurgitation ≥3+ were retrospectively selected and included in the present analysis. Patients with aortic regurgitation >2 were excluded from the analysis except for one case where rescue TAVI was performed to treat cardiogenic shock secondary to acute aortic regurgitation. Outcomes data for TAVI and MitraClip were reported according to the Valve Academic Research Consortium (VARC) criteria [4, 5].

All TAVI and MitraClip procedures were performed in a hybrid operating theatre under general anaesthesia, mechanical ventilation, with transoesophageal two- and three-dimensional echocardiographic and fluoroscopy guidance.

Echocardiography was performed prior to the procedure, during the procedure, immediately after it and at follow-up. The severity of mitral regurgitation, aortic stenosis, and aortic regurgitation were graded in accordance with the American Society of Echocardiography [6]. Grading criteria for postprocedural mitral regurgitation were adapted to the quantitative assessment of severity of mitral regurgitation in percutaneous mitral valve repair, as reported by Foster and co-workers [7].

When required, comparison between pre-procedural and post-procedural recorded values was performed by means of paired Student’s t‑test and chi-squared test, whenever applicable. Significance was stated with a *p* < 0.05. Follow-up estimated survival and freedom from 1‑year endpoints were calculated using Kaplan-Meier statistics.

## Results

From February 2010 to December 2013, 14 patients underwent staged percutaneous treatment of mitro-aortic pathology (TAVI followed by MitraClip). Demographic/comorbidity data are summarised in Table 1. The average interval from TAVI to mitral valve repair was 101 ± 12 days. A total of 12 patients (85.7%) had moderate to severe MVR at the time of TAVI. The remaining patients (2; 14.3%) had severe MVR. In the majority of patients (10/14; 71%) MVR had a secondary aetiology. According to the VARC criteria [4, 5] device/procedural success for TAVI was 100% and 92.9% (13/14) for MitraClip. In one patient with moderate to severe primary MVR, single MitraClip detachment was confirmed few days after implantation. The patient underwent mitral valve replacement 31 days after MitraClip due to symptomatic severe MVR. No patients developed a mitral valve gradient >5 mm Hg after MitraClip therapy and gradients passed from 2.1 ± 0.8 to 3.4 ± 0.5 mm Hg (*p* = 0.7). Early safety was 100% for TAVI and 85.7% for MitraClip.

**Table 1:** Preoperative, perioperative, and follow-up results in 14 patients undergoing staged transcatheter aortic valve implantation (TAVI) and MitraClip implantation

Age, years ± SD	78 ± 5
Male gender	8 (57.1%)
EuroScore II	19.0 ± 12.0
Prior acute decompensated HF, *n* (%)	14 (100)
NYHA class III/IV	13 (92.9%)
Coronary artery disease, *n* (%)	8 (57.1%)
*Procedural data TAVI*	
Total procedure time, min (mean ± SD)	96.8 ± 19.6
Fluoroscopy time, min (mean ± SD)	16.0 ± 4.8
Medtronic Corevalve, *n* (%)	9 (64.3%)
Edwards Sapien, *n* (%)	4 (28.5%)
Lotus Valve System, *n* (%)	1 (7.1%)
*Procedural data MitraClip*	
Total procedure time, min (mean ± SD)	154.6 ± 47.8
Fluoroscopy time, min (mean ± SD)	24.6 ± 12.5
– 1 clip, *n* (%)	4 (28.6%)
– 2 clip, *n* (%)	6 (42.9%)
– 3 clip, *n* (%)	4 (28.6%)
**In-hospital and 1‑year clinical results**	
Follow-up days	664 ± 651
Death	4 (28.6%)
– In-hospital, *n* (%)	1 (7.1%)
– 30-day, *n* (%)	1 (7.1%)
– 1 year, *n* (%)	4 (28.6%)
New permanent pacemaker	3 (21.4%)
Myocardial infarction, *n* (%)	0 (0)
Stroke, *n* (%)	0 (0)
Major bleeding, *n* (%)	1 (7.1%)
NYHA in surviving patients:	
– I – II – III	1 (7.6%) 8 (61.5%) 4 (30.7%)
Cardiac hospitalisation, *n* (%)	4 (28.6%)
Recurrent MR grade 3, *n* (%)	3 (21.4%)
Significant MV stenosis, *n* (%)	0 (0)
Significant AS/AR, *n* (%)	1 (7.1%)

*SD* standard deviation, *HF* heart failure, *NYHA* New York Heart Association, *MR* mitral regurgitation, *MV* mitral valve, *AS/AR* aortic stenosis/aortic regurgitation

More specific morbidity and mortality data are summarised in Table 1. One patient (7.1%) died in-hospital after MitraClip. At late follow-up, two additional patients with secondary MVR re-developed moderate to severe MVR. Both patients had been implanted with a single MitraClip and had preprocedural severe MVR. No patients developed mitral valve stenosis. In total four patients died: one 4 days after intervention due to COPD and right heart failure (mitral regurgitation was 2+ after clip); one after 32 days from cerebral haemorrhage; one after 194 days from pneumonia/sepsis, and one after 194 days from diabetic coma.

Follow-up functional class in survivors is reported in Table 1. More specifically, after TAVI, 7 (50%) patients were in NYHA class III and 7 (50%) in class IV. At follow-up after MitraClip therapy none of the surviving patients were in NYHA IV and there was a non-significant trend for NYHA class improvement (from NYHA III/IV 100% to NYHA III/IV 30.7%; *p* = 0.2). Estimated 1‑year survival was 66.5%. Freedom from 1‑year endpoint (death, stroke, major bleeding, myocardial infarction, and cardiac re-hospitalisation) was 57.9% (Table 1).

## Discussion

Combined TAVI and percutaneous mitral valve repair has been recently proposed to treat patients with prohibitive risks [8–10]. In our view, a staged approach seems the only logical and reasonable alternative. In fact, patients with compromised heart function and multiple episodes of heart failure may benefit from an initial handling with TAVI to stabilise the clinical condition and evaluate future evolution of MVR and heart failure symptoms. TAVI in the presence of severe MVR will not preclude consequent treatment of the mitral disease.

Just to clarify our personal experience, in the same time frame a total of 31 patients were treated with TAVI while having concomitant severe MVR. We decided to first approach the valve pathology that had the strongest impact *quoad vitam*. In the majority of patients an improvement in symptoms, most probably resulting from an increase in systolic left ventricular function, has been documented after sole TAVI. Because a significant reduction of MVR is not consistently reported, our indication for further treatment of MVR is mainly based on symptom recurrence in spite of maximal medical treatment. The 14 patients discussed were readmitted for persistent symptomatic moderate to severe and severe MVR. They represent the entirety of patients who showed no improvement at all in their clinical picture after sole TAVI as a result of persistent MVR [8].

In these complex patients, TAVI is reproducible with a high rate of success. Compared with TAVI, percutaneous MV repair carries a heavier burden of periprocedural and follow-up failures mainly due to recurrent MVR, resulting from the constant leaflet tethering that is often present in secondary MVR. Although this subgroup of patients with persistent severe mitral regurgitation after TAVI is often proposed for MitraClip therapy, outcomes should be analysed critically, in the light of the tremendous additional clinical and budgetary burden involved. In fact, as recently confirmed in the German TRAMI registry, the estimated 1‑year survival of these patients may be lower than 50% [11].

In our previously published experience with transcatheter management of double valve disease, 6‑month follow-up findings showed no recurrent symptomatic severe MVR [8]. With extension of follow-up, results seem less encouraging. In spite of acute success in the treatment of severe MVR, the 1‑year estimated major adverse cardiac and cerebrovascular event rate and heart failure re-hospitalisation rate after the MitraClip procedure is greater than 40%. Almost a fourth (21%) of our patients experienced recurrent severe MVR which led, in each case, to recurrence of symptoms and re-hospitalisation. In truth, all patients had already experienced at least one episode of heart failure requiring hospitalisation before MitraClip therapy and 70% of them had secondary MVR associated with coronary artery disease.

In light of these findings, the real benefits of an additional percutaneous mitral valve treatment in patients previously submitted to TAVI should be investigated in a prospective fashion and compared with maximal medical treatment. We believe that in patients with secondary MVR it is unreasonable to expect consistent long-term results by application of a single MitraClip.

In the near future the availability of additional percutaneous tools may represent an alternative to the MitraClip and to conventional surgery. Implantation of a percutaneous mitral valve ring could be hypothesised, as sole procedure or in combination with the MitraClip. These armamentaria could be applied in an escalating fashion, with the aim of preserving the native mitral valve anatomy. In patients with advanced changes in mitral valve/left ventricular geometry and function accompanied by primary degeneration of the mitral valve, a totally percutaneous mitral valve replacement should be auspicated. In any case, any further evolution of the MitraClip technology will inevitably bring an increase in perioperative instrumentation with added intraprocedural and postprocedural risks.

In conclusion, although our case series documents that a staged and fully percutaneous treatment of mitro-aortic pathology is technically feasible even in very comorbid patients, the recurrence of symptoms leading to repeat hospitalisation is high, and is usually secondary to further cardiac decompensation leading to MVR exacerbation. The limited size of our present series, which remains one of the largest ever presented, does not allow us to chase determinants of follow-up outcome. We can reasonably hypothesise that in the future 1) technical ameliorations to address percutaneously MVR and 2) earlier referral for treatment, could contribute improving the mid- and long-term outcomes of such complex patients.
